# Pretransplantation use of the second-generation tyrosine kinase inhibitors has no negative impact on the HCT outcome

**DOI:** 10.1007/s00277-015-2457-1

**Published:** 2015-07-29

**Authors:** Agnieszka Piekarska, Lidia Gil, Witold Prejzner, Piotr Wiśniewski, Aleksandra Leszczyńska, Michał Gniot, Mieczysław Komarnicki, Andrzej Hellmann

**Affiliations:** Department of Hematology and Transplantology, Medical University of Gdansk, Debinki 7, 80-952 Gdansk, Poland; Department of Hematology and Bone Marrow Transplantation, Poznan University of Medical Sciences, Szamarzewskiego 84, 60-569 Poznan, Poland; Department of Endocrinology and Internal Diseases, Medical University of Gdansk, Debinki 7, 80-952 Gdansk, Poland

**Keywords:** Allogeneic hematopoietic cell transplantation, Chronic myeloid leukemia, Second-generation tyrosine kinase inhibitors, Dasatinib, Nilotinib

## Abstract

**Introduction:**

Allogeneic hematopoietic cell transplantation (HCT) was a standard therapy in chronic phase (CP) chronic myeloid leukemia (CML). As a result of the effective therapy with tyrosine kinase inhibitors (TKI), HCT was shifted to defined clinical situations. We present the results of observational prospective analysis of 28 CML patients undergoing HCT after exposure to, at least, two lines of TKI (including dasatinib and/or nilotinib), with respect to response, overall survival (OS), treatment toxicity, graft versus host disease (GVHD), and progression/relapse incidence.

**Results:**

All the patients but one engrafted with median time 19 days. OS for patients in CP1 and CP2/accelerated phase (AcP) were 92.9 and 85.7 %, respectively. Six patients allotransplanted in blast crisis (BC) CML died early after HCT.

Eighteen patients achieved deep molecular remission (MR^4.5^ or MR^4.0^). Relapse incidence was 29.6 %. Median time to progression (TTP) differs significantly depending on the CML phase prior to HCT, the best response achieved after HCT and development of chronic GvHD.

NRM yielded the values 7.1, 12.5, and 50 % in CP1, CP2/AcP, and BC, respectively. Fatal outcome, due to veno-occlusive disease (VOD), was observed in two (7 %) patients. In five (17.9 %) patients, mild or moderate VOD was observed with no negative impact of preceding therapy with TKI2. Acute GvHD was diagnosed in 25.9 % of patients, while chronic GvHD developed in 42.9 % of individuals.

**Conclusion:**

Pretransplantation therapy with TKI2 in CP CML is safe and reasonable. In BC, the optimal approach before HCT is to reduce the leukemic burden and achieve subsequent CP.

## Introduction

Allogeneic hematopoietic cell transplantation (HCT) was historically a standard curative therapy for patients with chronic phase (CP) chronic myeloid leukemia (CML). As a result of the novel highly effective therapy with tyrosine kinase inhibitors (TKI), the use of HCT was shifted to defined clinical situations. Following the European LeukemiaNet (ELN) guidelines, patients with Philadelphia positive (Ph+) CML diagnosed in the advanced phase (accelerated phase (AcP); blast phase/crisis (BC)) or resistant to imatinib or second-generation TKI (TKI2), should be qualified to HCT [1]. A number of published data shows no serious negative impact of imatinib mesylate therapy prior to HCT [2–5], but only few studies concerning the outcome of HCT in TKI2-resistant patients are currently available [6].

Non-relapse mortality (NRM) and organ toxicity in patients receiving TKI2 before transplantation are of interest. Based on the toxicity profile of both dasatinib and nilotinib, development of hepatic veno-occlusive disease (VOD), cardiac toxicity, as well as immune dysfunctions or delayed engraftment can be expected [7, 8]. The long term outcome of TKI2-resistant patients remains another important issue. Potentially poorer outcome of patients pre-treated with TKI2 may result from the selection of more aggressive clones of malignant cells with additional molecular aberrations, promoting their proliferation and survival abilities. Moreover, TKI2-resistant patients are usually transplanted in more advanced phases of the disease or are simply older, when they finally enter the procedure of HCT. Additional problems are related to the late toxicity of HCT and chronic GvHD, significantly affecting the quality of life.

In this study, we present the results of observational prospective analysis of CML patients undergoing allogeneic hematopoietic cell transplantation after exposure to TKI2, as the second-line therapy, with respect to response, overall survival, treatment toxicity, GVHD and progression/relapse incidence.

### Patients and methods

#### Patients

The eligible subjects were patients undergoing allogeneic hematopoietic cell transplantation with CML diagnosis, after pretreatment with imatinib mesylate and one or more TKI2. The therapy with TKI2 was discontinued at least 2 days prior to the conditioning regimen. Treatment was provided in two transplant centers, between February 2008 and November 2013.

The study was approved by the Bioethical Committee of Medical University of Gdansk and was conducted in accordance with the Declaration of Helsinki.

#### Definitions

Chronic phase CML was diagnosed according to ELN criteria when blasts in peripheral blood (PB) and bone marrow (BM) were <15 %, basophilia did not exceed 20 %, and there was no evidence of extramedullary involvement. Accelerated phase was defined by blasts between 15 and 29 %, basophilia exceeding 20 %, persistent thrombocytopenia (<100 G/l, not related to the treatment) or thrombocytosis unresponsive to the therapy or any cytogenetic evidence of clonal evolution. Blast phase/crisis was diagnosed when there were 30 % or more PB or BM blasts, or the extramedullary blast proliferation occurred.

Molecular monitoring was performed according to the ELN guidelines with the RQ-PCR method [9–11]. Deep molecular response MR^4.5^ was defined as a 4.5-log reduction from the baseline (≤0.0032 % BCR-ABL [IS]) or a disease undetectable in complementary DNA (cDNA) with the amount of the transcript ABL ≥32.000. Deep MR^4.0^ was defined as a 4-log reduction from the baseline (≤0.01 % BCR-ABL [IS]) or a disease, which was undetectable in cDNA with the amount of the transcript ABL ≥10.000. Major molecular response (MMR) was determined when there was a 3-log reduction from the baseline (≤0.1 % BCR-ABL [IS]). Minor molecular response (MiMR) was the response below MMR.

Progression/relapse of CML after HCT was defined by any hematologic, cytogenetic, or molecular evidence of the disease, requiring the additional treatment after HCT (e.g., TKI re-administration and/or donor lymphocyte infusion (DLI)).

Engraftment was achieved when an absolute neutrophil count was greater or equal to 0.5 G/l, and it was the first of three consecutive days with this value.

Acute graft versus host disease (GvHD) was diagnosed according to IBMTR criteria [12, 13]. The diagnosis of chronic GvHD was based on NIH consensus criteria [14].

Hepatic veno-occlusive disease (VOD) was diagnosed with occurrence of at least two modified Seattle criteria: hyperbilirubinemia (>2 mg/dl), hepatomegaly with right upper quadrant abdominal pain, and >2 % weight gain due to fluid retention [15].

NRM was defined as any case of death without symptoms of relapse.

### Statistical analysis

Differences in median values between groups were analyzed with the Mann-Whitney *U* test. Correlations were analyzed using the Spearman’s rank test. Stratified Kaplan-Meier curves were applied to evaluate association between patient characteristics and the outcomes. Log-rank test was used to compare the curves. Since for most of the variables the median survival was not achieved, we reported the mean survival time restricted to the longest follow-up time. For most of the variables, the largest observed analysis time was censored and underestimation of the restricted mean survival time was expected. Multivariate analysis was performed using the Cox regression. *P* values <0.05 were considered statistically significant. Calculations were performed using the Stata 13.1 statistical software (StataCorp, TX, USA).

## Results

We prospectively analyzed the data of 28 patients treated with the second generation TKI (dasatinib, nilotinib, or both in sequence) before HCT. None of the studied patients, including those with BC, received conventional chemotherapy prior to HCT. Their characteristics are presented in Table 1. Patients were followed-up up to February 2014.

**Table 1:** Patient characteristics

Characteristics	Value
Sex, no. (%)	
Male	13 (46)
Female	15 (54)
Median age at HCT (range)	48 (29–68)
Second-generation TKI (TKI2), no. (%)	
Dasatinib	15 (53)
Nilotinib	5 (18)
Both	8 (29)
Disease phase at diagnosis, no. (%)	
Chronic phase (CP)	20 (72)
Accelerated phase	4 (14)
Blast phase (crisis)	4 (14)
Disease phase at TKI2 treatment, no. (%)	
Chronic phase	20 (72)
Accelerated phase	2 (7)
Blast phase (crisis)	6 (21)
Disease phase prior to HCT, no. (%)	
First chronic phase (CP1)	14 (50)
Accelerated phase or second CP (CP2)	8 (29)
Blast phase (crisis)	6 (21)
TKI2 therapy duration prior to HCT	
Median month (range)	14 (2–60)
≥12 months, no. (%)	13 (46)
<12 months, no. (%)	15 (54)
Donor type, no. (%)	
HLA-identical sibling	10 (36)
Matched unrelated	18 (64)
Conditioning regimen, no. (%)	
MAC (BuCy; TBICy)	15 (54)
RIC (FluMel; FluBu)	13 (46)
Graft type, no. (%)	
PBSC	24 (86)
BM	4 (14)
CD34 cells × 10^6^/kg, median (range)	
PBSC	4.1 (2.2–11.2)
BM	2.4 (1.5–3.0)
Immunosuppression, no. (%)	
MTX + CsA	10 (36)
ATG + MTX + CsA	18 (64)

*HCT* hematopoietic cell transplantation, *TKI2* second-generation tyrosine kinase inhibitors, *MAC* myeloablative conditioning, *RIC* reduced-intensity conditioning, *PBSC* peripheral blood stem cells, *BM* bone marrow, *MTX* methotrexate (days +1,+3, +6, +11), *CsA* cyclosporin, *ATG* antithymocyte globulin

### Engraftment

All the patients, except for one in BC, engrafted with median time 19 days (13–43 days). The duration of TKI2 therapy prior to HCT had no impact on the time of engraftment (*p* = 0.41).

#### Response

Eighteen patients achieved MR^4.5^ or MR^4.0^ with median time 1.8 months after HCT, and this response was maintained in 14 alive patients. Five patients achieved MMR, as the best molecular response, with median time 1.1 months after HCT, and this group included one patient in early post-transplant period, one who died due to aGvHD and three patients who lost this response. Four patients achieved responses below MMR (MiMR) and all of them died, due to relapse or aGvHD.

The data concerning the time to achieve the best molecular response were available for 27 patients. The median value was 1.8 months after HCT and did not depend on patients’ gender (*p* = 0.4), age (*p* = 0.98), type of a donor (*p* = 0.23), and the source of hematopoietic cells (*p* = 0.25). Median time to achieve the best molecular response was the shortest for patients in CP1 (1.1 months) and the longest in BC CML at HCT (2.4 months) but the trend was not statistically significant (*p* = 0.35). The best molecular response was not influenced by aGvHD occurrence (*p* = 0.52) or cGvHD development (*p* = 0.96).

#### Survival

**Fig. 1:**
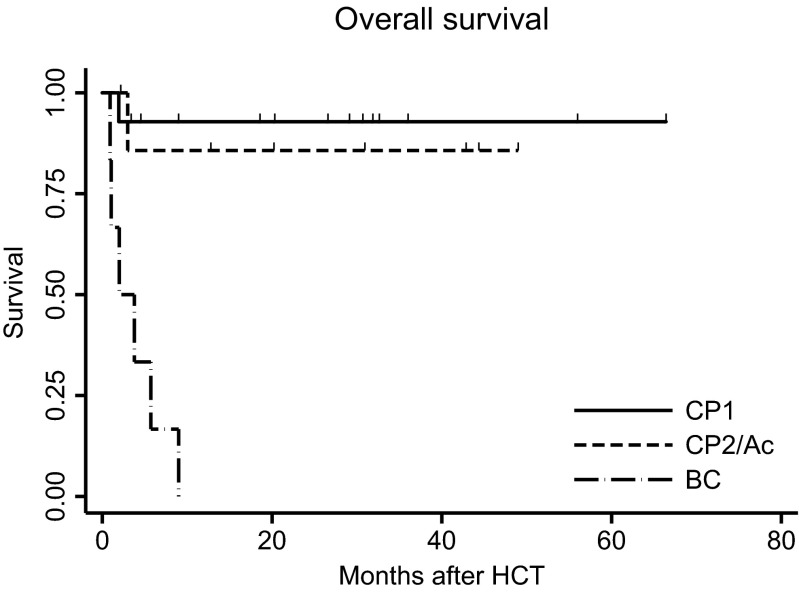
Impact of CML phase on overall survival (OS) after HCT. *CP1* first chronic phase, *CP2* second chronic phase, *Ac* accelerated phase, *BC* blast crisis/phase

During median 19 months (3–66) of follow-up, 19 patients survived. Deaths were related to VOD, aGvHD and relapse. Since all the deaths occurred within the first year after HCT, the 1- and 3-year observed survival rates were identical and yielded a value of 71.4 %. OS did not depend on patients’ gender (*p* = 0.97), age (*p* = 0.11), type of a donor (*p* = 0.97), and the source of hematopoietic cells (*p* = 0.83) but was strongly influenced by the CML phase prior to HCT (*p* = 0.0001), Fig. 1.

Crude OS estimates significantly depended on the best molecular response achieved after HCT; however, the result was confounded by the CML phase at HCT. After adjustment for the confounder, patients with deep MR had better prognosis, but the effect was not statistically significant (HR = 0.1; *p* = 0.08).

Kaplan-Meier estimates of OS for patients in CP1 and CP2/AcP were 92.9 and 85.7 %, respectively. In the group of 14 patients with CP1 one patient died due to acute GvHD (grade IV). Among eight patients with more advanced phase (CP2 or Ac): one died because of aGvHD (grade IV) and one allotransplanted from the family donor relapsed early in BC CML.

Six patients were allotransplanted in BC. All of them died in the early post-transplant period: three due to relapse, one as a result of fatal acute GvHD, one because of VOD and one due to multiorgan failure (MOF)/VOD.

#### Time to progression

Median time to progression (TTP) in the whole study group has not been achieved yet, while the mean time has reached 45 months. Median TTP in the group of patients, who lost their molecular response was 5 months.

**Fig. 2:**
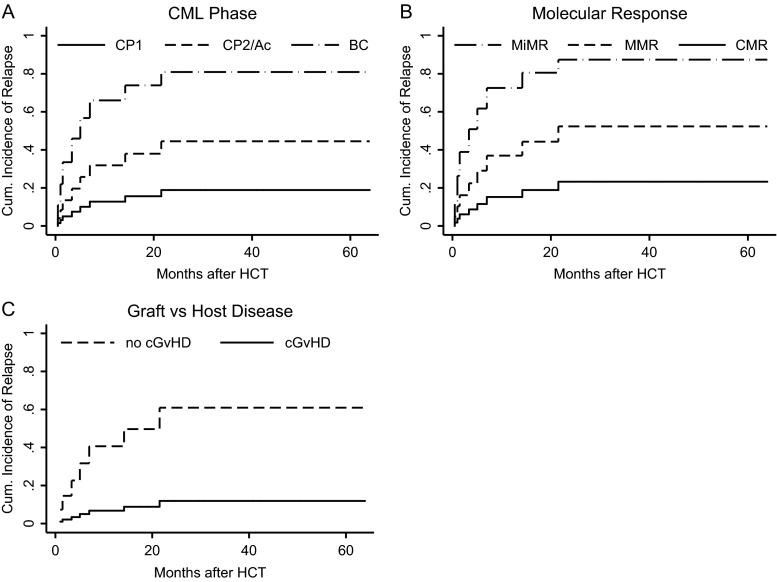
**a**–**c** Impact of clinical factors on the incidence of relapse. Cumulative rates of relapses were estimated according to the competing risk method. Non-relapse mortality was evaluated as a competing risk. *CP* chronic phase, *Ac* accelerated phase, *BC* blast crisis/phase, *CMR* complete molecular response, *MMR* major molecular response, *MiMR* minor molecular response

TTP did not depend on patients’ gender (*p* = 0.63), age (*p* = 0.98), type of a donor (*p* = 0.22), the source of hematopoietic cells (*p* = 0.55), and occurrence of aGvHD (*p* = 0.55) but differs significantly depending on CML phase prior to HCT (*p* = 0.046), Fig. 2a. TTP also differs depending on the level of response achieved after HCT (*p* = 0.009), Fig. 2b. Moreover, TTP was significantly influenced by the development of cGvHD (*p* = 0.038), Fig. 2c.

#### Relapse incidence

Relapse after HCT was seen in eight patients (29.6 %). The incidence of relapse in CP1, CP2/Ac, and BC was 21.4, 12.5, and 50 %, respectively. Altogether, three molecular relapses and five hematological relapses were diagnosed. Hematological relapse was a direct cause of death in three cases. Those patients were in BC prior to transplantation and had achieved at most MMR after HCT. Among the remaining four relapsed patients, three were transplanted in CP1 and one in CP2. All four patients received RIC prior to HCT. They were qualified to DLI-based therapy: three due to molecular relapses and one due to hematological relapse (combined treatment with DLI and dasatinib). In all of them deep MR is maintained until the end of follow-up.

#### Treatment toxicity and GVHD

Total incidence of VOD was 25 % (seven patients) in the study group. Fatal outcome due to severe transplant-related toxicity was seen in two (7.1 %) patients, both in BC: hepatic VOD and MOF/VOD. In five (17.9 %) patients, mild or moderate VOD was observed with complete recovery after conventional treatment, including two patients in BC and three patients in CP1/CP2/Ac phase CML. The incidence of VOD in BC and CP1/CP2/Ac phase was 66.7 and 13.6 %, respectively. A prolonged therapy with TKI2 had no influence on VOD occurrence. Incidence of VOD development decreased along with the duration of TKI2 treatment (*p* = 0.019).

Acute graft versus host disease (aGvHD) was diagnosed in 7 out of 27 patients (25.9 %) including grades I–II in four patients (11 %). It was established in one (3.7 %) patient in CP1, three (11.1 %) patients in CP2/Ac, and three (11.1 %) patients in BC CML. Median survival of patients with aGvHD grade IV was 3 months.

Among 21 patients with the follow-up period exceeding 100 days after HCT, chronic GvHD (cGvHD) developed in nine (42.9 %) patients, including one person with cGvHD induced by DLI. In five (21 %) mild and in four cases, moderate cGvHD was observed. Chronic GvHD had no impact on OS (*p* = 0.18).

NRM reached 7.1 % for CP1, 12.5 % for CP2/AcP, and 50 % for BC and included deaths caused by VOD/MOF and GvHD-related complications.

## Discussion

The therapy with tyrosine kinase inhibitors has been a standard care for patients with CML for more than one decade; that is why data concerning the results of HCT performed nowadays in TKI-naive individuals are no longer available. Due to the advance in post-transplant care, our results cannot be compared reliably to a historical group, prior to the era of TKI, but might be compared to the published data concerning the imatinib-pretreated group of patients. The concept to compare data comes from the similarities in the toxicity profile of imatinib and TKI2.

The clinically important finding of our research is a lack of severe hepatic VOD and engraftment failure that could potentially complicate the use of second-generation TKI prior to HCT in a group of patients in early (CP1) or advanced (CP2 and AcP) phase of CML. The therapy with dasatinib or nilotinib in our study has been discontinued before the conditioning regimen started, the same as imatinib in published studies. Zaucha et al. [5] presented the results of transplantation in the group of 30 patients receiving imatinib prior to HCT, including 26 patients with CML. The group characteristics differ with the older median age (48 vs 32 years), a smaller number of patients transplanted from BM and a higher proportion of patients in BC CML in our study group. Graft failure was reported in two patients in the imatinib group and in one patient in the TKI2 group, while median time to engraftment after PBSCT was comparable in both groups. To confront hepatotoxicity, 23 % of patients in the imatinib group experienced severe hyperbilirubinemia including 10 % who met VOD criteria, while in our TKI2 group, 25 % developed VOD, including 7.1 % with fatal outcome, however observed in patients with BC CML. Patients in CP1/CP2 in our study had VOD incidence about 14 %, that is comparable to cumulative incidence of VOD recently reported as about 13.8 % using Seattle criteria [16], and there was no VOD/MOF-related death in this group. Moreover, a prolonged TKI2 therapy, reducing the leukemia burden, was associated with lower incidence of hepatic toxicity. Therefore, the strategy to continue TKI2 treatment as long as it is necessary to find a donor is a reasonable tactic and TKI2 tapering few days prior to conditioning appears to be the safe approach.

In contrast to transplantation performed in CP1, CP2, or AcP phase, BC CML increases significantly the risk of severe complications and relapse after HCT, which is in line with data reported by other authors [17, 18]. It is essential to reduce the load of leukemic cells by TKI and/or chemotherapy to the level of the second or subsequent CP to achieve the long-lasting remission. In contrast to high relapse rate in patients in BC, among seven patients transplanted in CP2 only one patient died due to relapse, one had molecular relapse successfully treated with DLI, and one died due to aGvHD grade IV in deep MR. With this thesis, we suggest not to postpone HCT in case of hematological response to TKI2 while waiting for the deeper molecular response and risking the clonal progression. Moreover, patients with BC at HCT should be attentively and frequently monitored after transplantation with the use of the most sensitive PCR-based tests to detect molecular progression or relapse. In this high-risk group of patients, the use of DLI with re-introduction of TKI, may prevent the disease progression and improve the outcome. It is well known that patients with relapse detected on the molecular level respond to DLI with higher remission rate compared to any more advanced disease [19, 20].

We also analyzed the incidence of acute and chronic GvHD since the immunomodulatory potency of TKI may determine the incidence and severity of GvHD. In case of imatinib, the suggested mechanism of immunosuppression is probably related to inhibition of T cell activation and dendritic cell development [21]. Similar data concerning nilotinib, but mainly based on in vitro studies, have been reported [22]. The efficacy but also toxicity profile of dasatinib is correlated with immunomodulatory activity related to the induction of monoclonal T/NK cell proliferation [23]. It is explained by a broad spectrum of inhibited kinases, including SCR-kinase. Dasatinib has also an ability to block T cell activity, proliferation, secretion, and TCR-signaling pathway [24–26]. The significance of these clonal lymphocytes in anti-leukemic surveillance and their ability to influence graft versus leukemia and GvHD have not been determined. In our TKI2 study group, the incidence of aGvHD was 25.9 % including 14.9 % in grade III-IV and was lower than that reported by Zaucha et al. [5].

Clinically important are encouraging results of survival and response after HCT achieved in patients with failure of TKI2 therapy. OS for 22 patients in CP1 and CP2/AcP were 92.9 and 85.7 %, respectively. Among them 18 patients achieved MR^4.5^ or MR^4.0^ with median time 1.8 months after HCT, and this response has been maintained in 14 alive patients. In four patients with relapse, deep MR was restored by DLI with/without TKI support. This confirms the important role of HCT in CML management.

We conclude that in the view of the curative potential of HCT and non-inferior survival of patients in CP CML with the pretransplantation TKI2 therapy, compared to the imatinib therapy, it is a safe and reasonable approach. Nonetheless, further studies are warranted to assess the individual influence of nilotinib and dasatinib on the transplant-related toxicity and the outcome of HCT. In blast crisis, the optimal approach before HCT is to reduce the leukemic burden. Achievement of subsequent CP seems to be sufficient to improve the outcome in this group of patients.
